# Endoscopic Distal Iliotibial Band Z-Plasty for Greater Trochanteric Pain Syndrome

**DOI:** 10.1016/j.eats.2024.103307

**Published:** 2024-11-16

**Authors:** Peter Joachim Bruun Thomassen, Jon Olav Drogset, Olav Foss

**Affiliations:** a Orthopaedics Department, St. Olav’s University Hospital, Trondheim, Norway; b Norwegian University of Science and Technology, Trondheim, Norway

## Abstract

Greater trochanteric pain syndrome (GTPS) includes idiopathic trochanteric pain, gluteus medius/minimus tendinopathy, and external snapping hip. If conservative treatment is unsuccessful, various open surgical procedures of the iliotibial band (ITB) at the level of the greater trochanter are available. Over the past few decades, endoscopic management of ITB and bursectomy for GTPS have been established as an alternative to open procedures. Better results and fewer complications are potential advantages of an endoscopic procedure. If surgery at the level of the greater trochanter fails, Z-plasty of the ITB at the level of the distal part of the femur may be considered. This operation relieves the ITB above the greater trochanter without exposing this area to further trauma. Traditionally, Z-plasty has been performed as an open procedure. This technical note describes an endoscopic method for the distal ITB Z-plasty.

Greater trochanteric pain syndrome (GTPS) is a condition characterized by trochanteric pain radiating from the greater trochanter and along the lateral part of the thigh, occasionally distal to the knee.^1^ For the idiopathic condition, the etiology is unknown; in other cases such as damage to the gluteus medius and minimus muscles and tendons or external snapping hip, the causal relationship is relatively clear.^2^ In the idiopathic condition or with degenerative damage to the gluteus medius/minimus, an acute trauma causal mechanism is unusual. These conditions exist mainly among middle-aged women.

External snapping hip occurs mainly in younger women.^3^^,^^4^ In addition to a thorough history, the investigation should consist of a conventional radiograph to rule out possible articular pathology or any exostoses on the greater trochanter.

Additionally, magnetic resonance imaging to look for any peritrochanteric edema or damage to the gluteus medius/minimus is performed.^5^ The clinical findings of distinct tactile tenderness over the greater trochanter and a positive Trendelenburg sign are indications of a gluteus medius/minimus rupture.^6^ Any differential diagnoses such as osteoarthritis of the hip joint and lumbar nerve root compression must be ruled out.^7^^,^^8^ If conservative treatment is still unsatisfactory, the patient can be offered operative treatment.

In the idiopathic state of GTPS, endoscopic division of the ITB is performed with simultaneous bursectomy at the level of the greater trochanter.^9^ In such a case, it is important to inspect the gluteus medius/minimus carefully so that any rupture can be sutured.^10^

A majority of patients experience improvement after primary surgery.^11^ For those who do not improve, a second intervention in the form of distal Z-plasty of the ITB can be considered. In trochanteric pain after hip arthroplasty, it is considered a relative contraindication to re-traumatize the peritrochanteric structures; therefore, a distal procedure of the ITB may be the method of choice.^12^ This procedure can also be performed on patients with external snapping hip.^13^

Iliotibial band (ITB) syndrome (runner’s knee) is a separate condition that can also be treated with a distal Z-plasty, or an endoscopic distal ITB lengthening.^14^^,^^15^ Traditionally, distal Z-plasty of the ITB has been an open procedure.

This technical note presents the Thomassen Z-plasty, an endoscopic procedure for distal Z-plasty of the ITB, which has advantages in terms of better results and fewer complications.^16^

## Surgical Technique

Surgery is performed under general or spinal anesthesia. The patient is placed in a lateral decubitus position with the distal part of the thigh washed and prepared.

**Fig 1:**
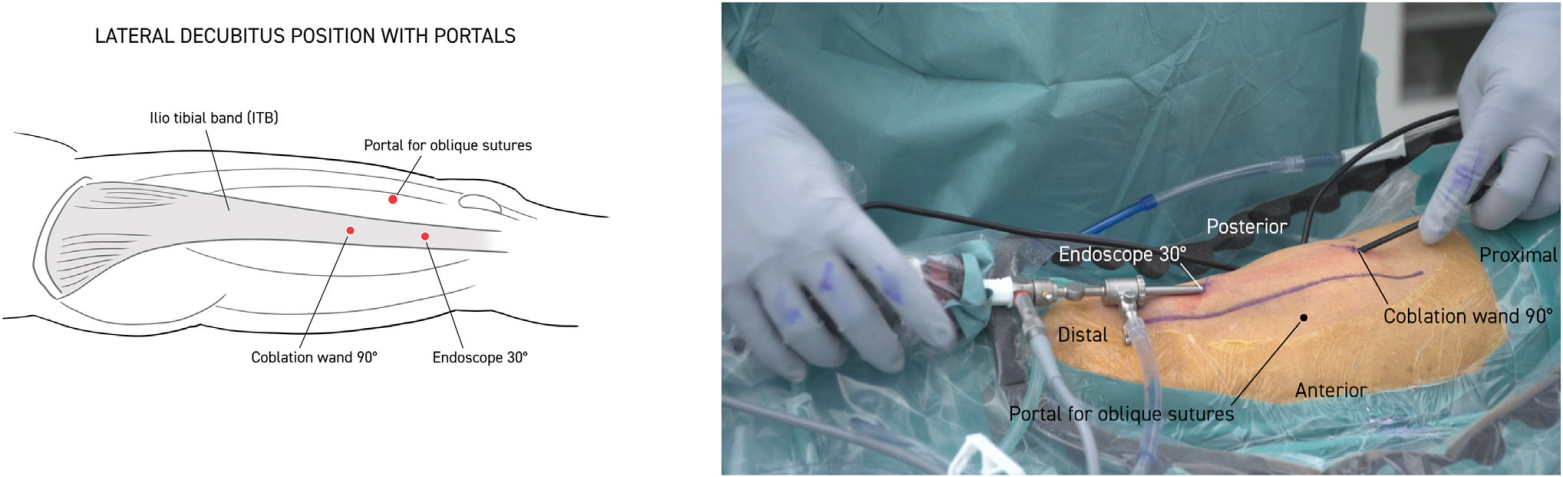
The patient is placed in a lateral decubitus position for procedure on the right thigh. Portal for the 30° endoscope and working portal for the 90° coblation wand, respectively, are 5 and 15 cm proximal to the lateral epicondyle of the femur. Anterior and posterior margins of the iliotibial band (ITB) are marked longitudinally with a pen (blue). Subcutaneous fatty tisse is removed with the coblation wand visualizing the ITB. Continuous hemostasis is performed.

**Fig 2:**
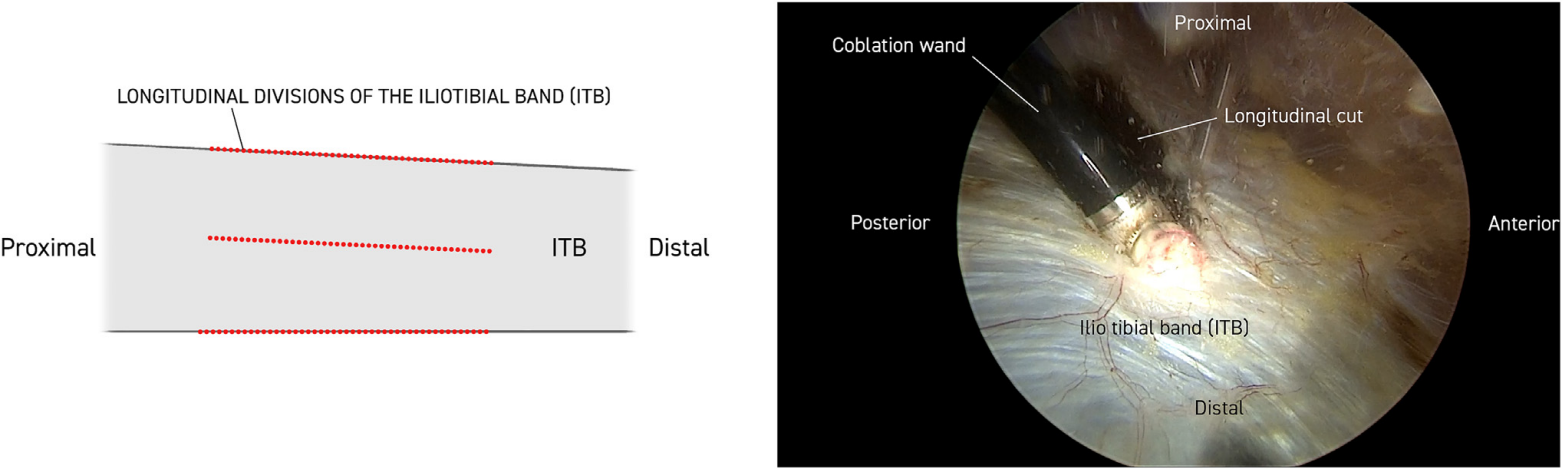
The iliotibial band (ITB) is divided longitudinally with the 90° coblation wand 3 times, first at the anterior margin, then at the posterior margin, and finally in the middle in the proximal-distal direction. Work is always conducted with the coblation wand facing outward, respecting the vastus lateralis muscle. Each cut is approximately 15 cm.

**Fig 3:**
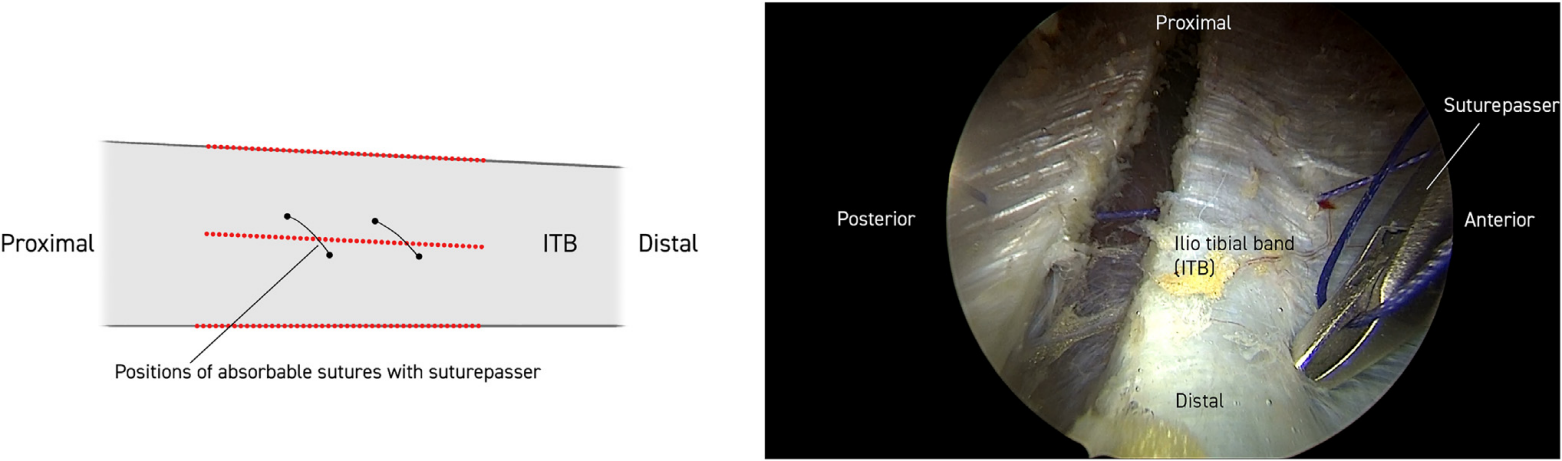
With a cannula, the level of the first of 2 single absorbable sutures is probed obliquely through the anterior to the posterior part of the iliotibial band (ITB). The first (proximal) suture is then passed through the 2 ITB strings with the help of a suture passer. The procedure is repeated for the other distal bevel.

**Fig 4:**
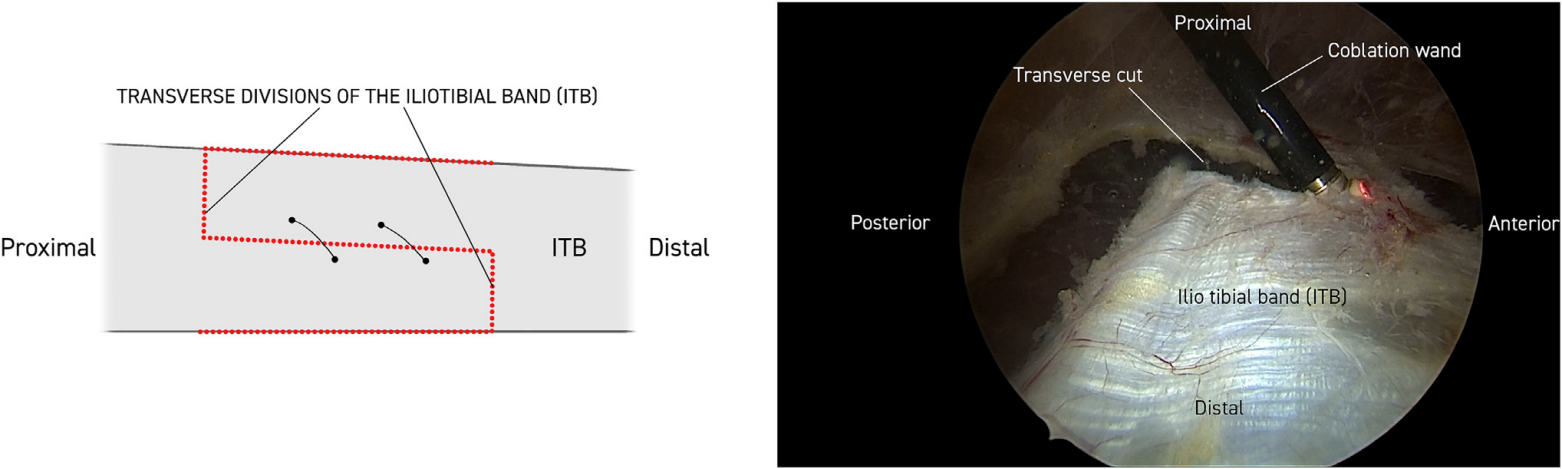
The iliotibial band (ITB) is divided transversely anterior-proximally and posterior-distally with the 90° coblator wand elongating the ITB approximately 3 cm.

**Fig 5:**
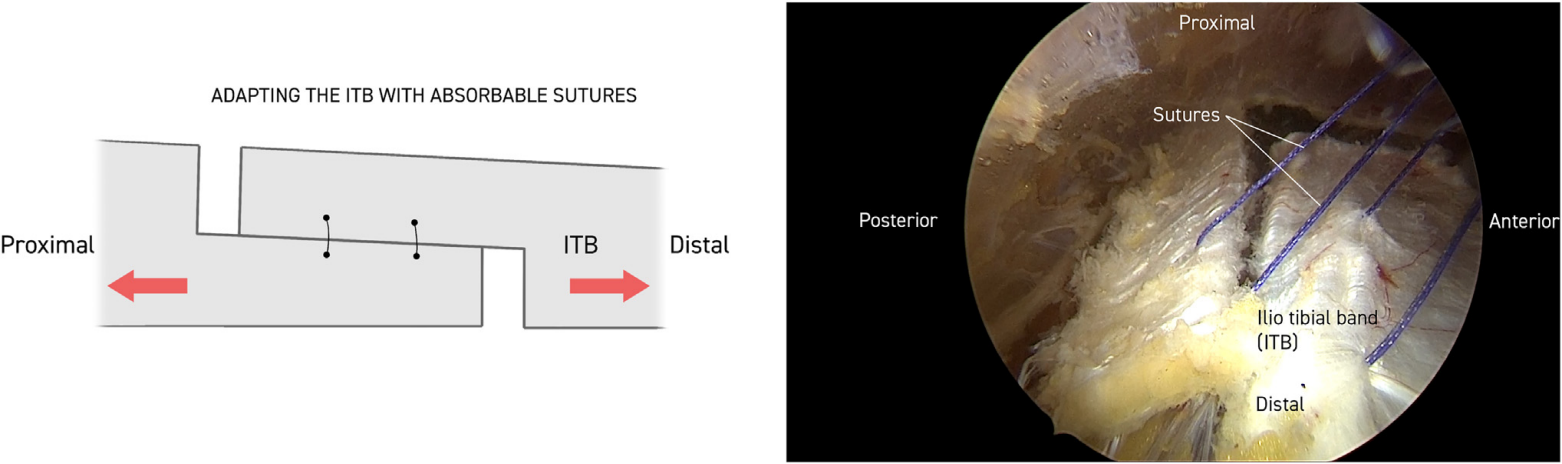
After longitudinal and transverse division of the iliotibial band (ITB), the sutures lie parallel and can be sutured with the help of a knotpusher and sliding knots. Final meticulous hemostasis is done with the 90° coblation wand and skin closure with 3 mattress sutures.

Twenty milliliters of Marcaine with adrenaline solution is injected subcutaneously. Anterior and posterior limitations of the ITB are delineated, and a 30° standard endoscope (Stryker) is introduced to the distal portal approximately 5 cm proximal to the lateral epicondyle of the femur. Approximately 10 cm proximal to this, a working portal is established, including a 90° coblator wand (Smith & Nephew Werewolf Coblation System) to remove subcutaneous fat tissue adherent to the ITB while assisting with hemostasis (Fig 1, Fig 2 and Fig 1, Fig 2). The ITB is then divided with the coblator wand longitudinally 3 times, first at the anterior margin, then at the posterior margin, and finally in the middle (Fig 2). With a cannula, the level of the first of 2 single absorbable sutures is then probed obliquely through the anterior to the posterior part of the ITB. The suture is then passed through the 2 ITB strings with the help of a suture passer (Arthrex Birdbeak 22°). The procedure is repeated for the other distal bevel (Fig 3). The ITB is then divided transversely anterior-proximal and posterior-distal with the coblator wand (Fig 4). The sutures then lie parallel and can be sutured with the help of a knotpusher (Smith & Nephew) and sliding knots (Fig 5). Hemostasis is secured, and finally the skin is closed using 3 mattress sutures (Video 1).

### Postoperative Rehabilitation

Free mobilization is permitted from the first postoperative day. Physiotherapy is identical to preoperative care with abduction and outward rotational exercises in the hip.

## Discussion

The treatment of GTPS, whatever the etiology, can be challenging, and given that the ITB is a prominent entity in the proximal part of the lower extremity, function and anatomy remains partially an enigma.^17^ This patient group has often undergone many conservative treatments. Not infrequently, patients are told that the condition has no surgical solution. However, research data indicate good to excellent results for reduction of pain and increased function with surgery.^9^^,^^11^ The largest volume consists of idiopathic pain in middle-aged women, followed by external snapping hip and finally gluteus medius/minimus ruptures. For the latter patient category, endoscopic suture of gluteus medius with specially made anchors has shown good results.^10^ For patients with idiopathic trochanteric pain, a direct trochanteric procedure is indicated to inspect the gluteus medius and minimus because this is often the cause of the pain. A degenerative or partial to full rupture may be found and should be addressed. Instead of treating an external snapping hip with a direct trochanteric procedure, the tight ITB can just as easily be released with a distal Z-plasty, and not infrequently the snapping will disappear immediately after the surgery.

In the case of trochanteric pain after hip arthroplasty, it is desirable to spare the trochanteric structures from the new trauma of a direct trochanteric operation, and instead offer a distal z-plasty. One can also consider endoscopic distal z-plasty for patients with ITBS.

For an experienced arthroscopist this procedure has a quick learning curve, and the pitfalls are few but include haematoma and seroma. As for most endoscopic procedures, this z-plasty also offers faster rehabilitation and better overall results (Table 1, Table 2 and Table 1, Table 2).

**Table 1:** Advantages and Disadvantages

Advantages	Disadavantages
Faster rehabilitation Fewer complications Minimally invasive Patients require scopic procedure when available Equal preparation time in the operating room Easy to convert to open procedure The only continuous cost is the absorbable suture and coblation wand	Few studies have been conducted on this method Learning curve

**Table 2:** Pearls and Pitfalls

Pearls	Pitfalls
Meticulous hemostasis Easy to identify anatomic landmarks No dangerous neurovaskular structures Negligible risk of skin necrosis	Hematoma Seroma

Current articles are mostly case reports and Level 4 studies.^18^ Further research is needed to provide more information about the pros and cons of these operating procedures.

## Disclosures

The authors (P.J.B.T., J.O.D., O.F.) declare that they have no known competing financial interests or personal relationships that could have appeared to influence the work reported in this paper.

## Supplementary Data

Video 1 In situ and animated video showing position of patient and surgeon and the iliotibial band Z-plasty.
